# Volumetric arc therapy for total scalp irradiation: case report for a recurrent basal cell carcinoma of the scalp

**DOI:** 10.3332/ecancer.2017.737

**Published:** 2017-05-11

**Authors:** Francisco Lozano, Naipy Perez, Alejandro Iglesias, Xiaodong Xu, Marco A Amendola, Michael Scott, Erich Companioni,, Beatriz E Amendola

**Affiliations:** Innovative Cancer Institute, 5995 SW 71st Street, South Miami, FL 33143, USA

**Keywords:** skin cancer, total scalp irradiation, Volumetric Arc Therapy, BCC

## Abstract

Total scalp irradiation may be used to treat numerous conditions including squamous and basal cell carcinomas. These conditions are relatively uncommon and patients are frequently treated with palliative intent. In this report, we describe a volumetric arc therapy technique using photon beams for curative intent in an 84 years old patient with recurrent basal cell carcinoma of the scalp. Dose was 50Gy (2Gy per session) to the planning target volume (PTV) followed by a 10 Gy boost to the macroscopic disease on the forehead. A custom made 1 cm superflab bolus helmet was used.

Toxicities only consisted of Grade-1 transient radiation dermatitis and alopecia. A sustained clinical response was observed at 6 months follow-up.

Volumetric arc therapy (VMAT) may offer an effective alternative modality to treat patients with very extensive scalp lesions as described in this case report.

## Introduction

Total scalp irradiation is a useful technique in the management of skin cancer and other types of malignancies; particularly in extensive tumours that are not amenable to surgery or when more standard radiation treatment fields overlap and create high-dose hot spots between the fields [1–14]. Fortunately, these conditions are relatively uncommon and patients are frequently treated with palliative intent due to the size and aggressiveness of the tumour.

Planning and delivering radiation treatment to a large region of the scalp is technically challenging due to the geometry of the head and the close proximity of the scalp to the brain, optical structures, and other nearby healthy organs at risk.

Since the scalp thickness is only 4–6 mm, electrons have traditionally been the modality of choice because of its high surface dose, rapid dose falloff and its finite range, with acceptable dose received by the brain resulting in only minimal or mild treatment-related toxicity. Nonetheless, electron treatments have presented difficulties because the scattering of electrons at oblique surfaces can create unusual dose distributions [3, 10]. Different techniques and approaches have been described in the literature such as: the 4 × 4 technique described by Mellenberg *et al*. [6] and the use of multiple matching fields described by Able *et al*. [3] Sagar and Pujara employed a similar technique with overlapping fields [11]. Electron techniques carry laborious treatment setup, special attention by well-trained physics staff and innate dosimetry challenges, such as dose heterogeneity across the target volume. More recently, alternative techniques, including brachytherapy, combinations of photon and electron fields, and IMRT, have been described in the literature [2, 5, 8]. Wojcika *et al*. compared lateral photon/electron plan, intensity modulated radiation therapy (IMRT) and high-dose rate (HDR) brachytherapy for total scalp irradiation [1]. Although IMRT provided the best dose homogeneity and coverage with acceptable doses to normal structures, HDR brachytherapy produced the most conformal plan, but the total dose delivered was limited by tolerance doses to the brain and eyes.

In this report, we describe a volumetric arc therapy technique using photon beams for curative intent in a patient with recurrent basal cell carcinoma of the scalp.

## Case report

**Figure 1.**
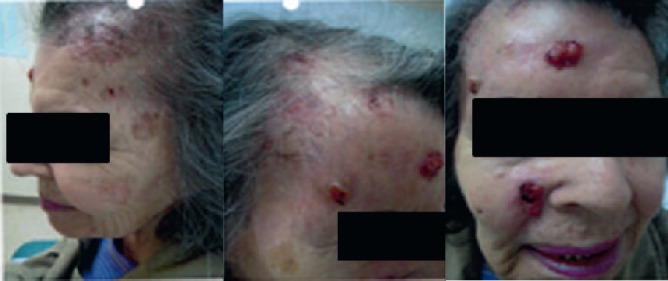
Before treatment: multiple lesions in the face and scalp are noted, consistent with extensive BCC.

An 84-year-old woman presented with a history of multiply recurrent basal cell skin cancers in the face and scalp, initially treated in an outside institution. The first documented occurrences date back to March 2010, at which time she underwent punch biopsy of a right upper lip tumour and shave biopsy of the left forehead and anterior scalp. Pathology confirmed that all three lesions were basal cell carcinoma (BCC). In 2014, she underwent Moh’s surgery for squamous cell carcinoma (SCC) of the right forehead. In March 2014, she had Moh’s surgery for BCC of the mid-forehead. In November 2015, she was referred to our institution for the consideration of radiation therapy to an extensive recurrent BCC of the left forehead and both temple areas extending into the hairline (Figure 1). The patient had previously been recommended treatment with chemo/immunotherapy for this lesion, but had declined any form of systemic therapy.

**Figure 2.**
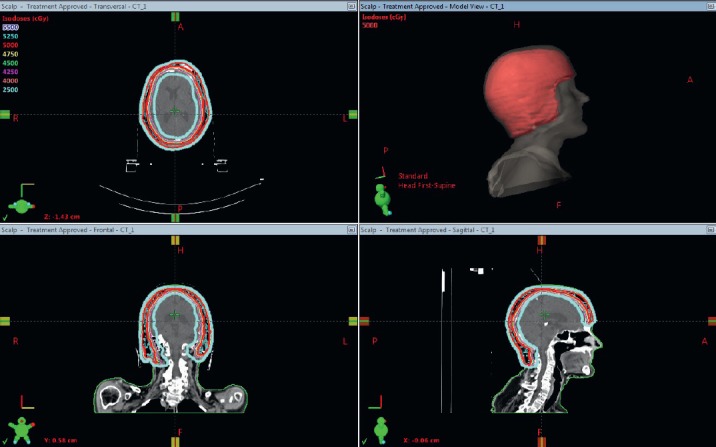
Patient was simulated and treated with a 1 cm custom made helmet bolus. Red isodose line is the Rx dose of 50 Gy for the first plan. Blue line, 25 Gy (50% of Rx dose) is shown, demonstrating excellent coverage with depiction of low dose to regions of the brain.

Simulation was performed with 3 mm cuts in a SOMATON™ Definition AS 64-slice CT scanner (Siemens Medical Systems®). The clinical target volume (CTV) included the skin surface to the depth of the cranium over the extent of the patient’s scalp (Figure 2). The planning target volume (PTV) was delineated as the CTV plus 3 mm margin. A 1 cm superflab bolus helmet was created.

The prescribed dose was 50 Gy (2 Gy per session) to the PTV followed by a 10 Gy conedown to the macroscopic disease on the forehead. The 1 cm bolus was used in both phases.

**Figure 3.**
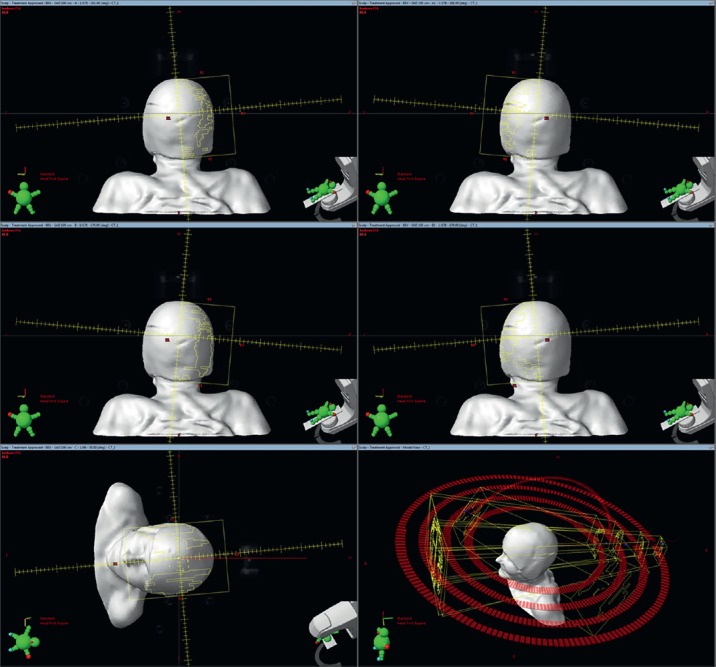
Showing beam arrangement: Top Beam A, A1, Middle Beam B, B1, Bottom Beam C (left) and C (right) all beams.

The treatment was delivered on the Edge™ unit (Varian Medical System®) using 6 MV X-rays. Cone beam CT was used prior to every fraction for daily position verification. Patient specific QA was performed using the ArcCHECK™ with verification system. The volumetric arc therapy (VMAT) plan was generated using four half-field arcs and one vertex. Details of each arc are described in Table 1 and shown in Figure 3.

The total number of monitor units (MU) per cGy was 4.9 MU/cGy (3.38 MU/cGy for the cone down phase). The treatment required an average of 15 minutes of machine time: 8 minutes for bolus (1 cm) placement and patient set-up, two minutes to obtain and analyse the CBCT images and 5 minutes for treatment delivery including the time for the couch rotation.

The conformity index to the PTV was 1.06.

**Figure 4.**
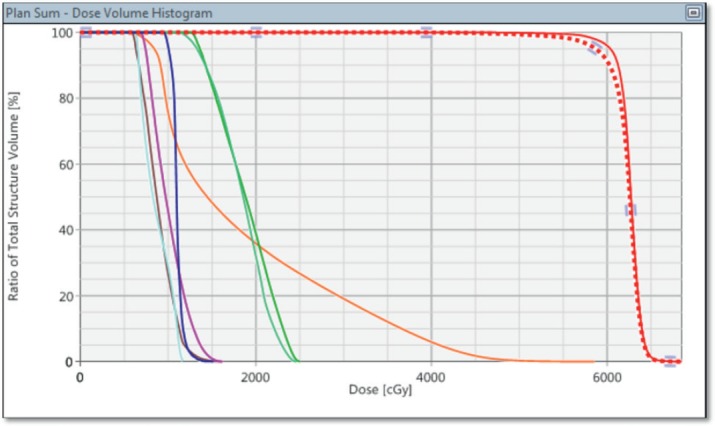
Dose volume histogram (DVH) of the composite plan showing 96 % of the CTV (Red) covered by 60 Gy. V30 of the brain (orange) is less than 20%. The remaining lines represent the dose to the optic nerves (green) with a maximum < 25 Gy, respectively, left lens (cyan) 11 Gy and right lens (magenta) 16 Gy (late formation of cataracts could be expected). The maximum doses to the optic chiasm (dark blue) and brainstem (brown) were less than 16 Gy.

The dose volume histogram (DVH) (Figure 4), demonstrated excellent coverage and a homogeneous dose within the target with acceptable dosage of the organs at risk. The homogeneity index: H index = [(D2%-D98%)/D50%] equals to 0.104. The mean dose to the brain in the composite plan is 1892 cGy.

During treatment, the patient only reported mild radiation dermatitis (Grade I). Mild pruritus was present during early follow-up, which resolved with topical medication.

**Figure 5.**
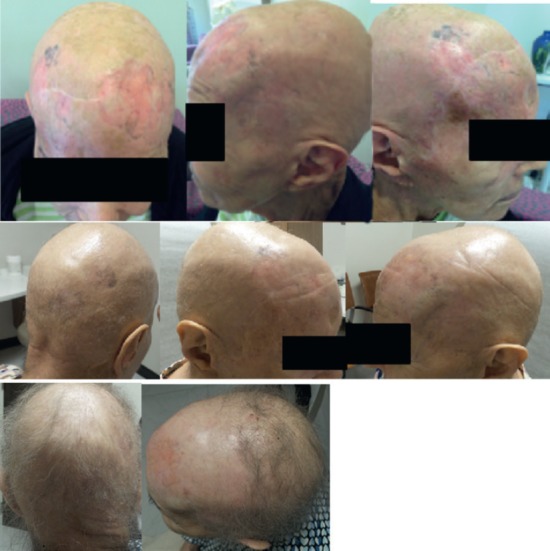
Top row: Mid treatment course: complete clinical response with mild dermatitis seen in patches. Grade-I pruritus was recorded clinically. Middle row: evaluation at second follow-up at six months shows complete response and resolution of the erythema. Bottom row: most recent follow-up at 1 year after treatment completion showing complete response and resolution of alopecia.

The patient was seen at 6 and 12 months after completing treatment with sustained clinical response. The only acute toxicity was alopecia which is resolving, as seen in her most recent follow up. No late toxicities were reported (Figure 5).

## Discussion

BCC and SCC of the skin are the most common neoplasm in the United States, with an incidence of over two million affected Americans annually [15]. The incidence of this common malignancy is rising rapidly, but the scalp is one the less common sites of appearance [15]. Usually, the first curative approach is surgery with or without post-operative radiation therapy. Definitive radiation therapy for tumours involving the entire scalp is infrequently used and total scalp irradiation is typically reserved for older patients with recurrent or extensive disease who are treated with palliative intent [4]. Radiotherapy of the scalp is a very complex technique, and at the moment, electron field radiotherapy and brachytherapy techniques are still considered the gold standard therapeutic modality [9].

Electron techniques require laborious setup, and several authors have documented its inferior homogeneity across the target volume, which could lead to inferior results [12, 13]. Brachytherapy, although reported to be the most conformal technique of all, demands an even more complex setup. There have been published reports of treatments using up to 29 catheters and 1028 dwell positions. This may be unacceptable in elderly patients when treatment time could last up to an hour, compared to an average of 15 minutes with VMAT as presented in this case report [4].

The advantages of VMAT treatment for scalp lesions include less technical demands of planning, faster daily set-up and treatment delivery, and simpler dose calculations that do not involve shielding as required in some electron techniques or the construction of surface molds that could affect reproducibility as required in some brachytherapy techniques. VMAT also offers excellent dose conformity and homogeneity compared with other treatment techniques and modalities.

The main disadvantage of VMAT treatment is the increased percentage of low-dose radiation received by the underlying brain, which is significantly higher than other techniques. However, this dose is still typically below the acceptable dose tolerance range and is often associated with no significant acute or late toxicity as was also reported in this case. Santos *et al*. recently reported a similar tangential VMAT technique, in which the tangential beam with collimating jaws protect dose sensitive organs at risk in close proximity to the planning target volume [9].

## Conclusions

VMAT may offer an effective alternative modality to treat patients with very extensive scalp lesions as described in this case report. Dose conformity and homogeneity with VMAT was superior to previous reports of other modalities described in the literature. It requires less complex planning, permits a more reproducible setup and demands less resources for the physicist’s calculations and quality assurance. Although increased regions of lower radiation dose to the brain still remains a concern for long-term toxicity, the doses achieved in this case report remain below acceptable dose tolerance constraints and resulted in no significant reported toxicity in a short follow up period for this patient.

## Figures and Tables

**Table 1.** VMAT plan using four half-field arcs and 1 vertex; 1 for 25 fractions of 2 Gy/fx.

Field	Couch position	Collimator	Gantry rotation	Monitor units
A	0	5	Full rotation CW	227
A1	0	-5	Full rotation CCW	227
B	0	5	Full rotation CW	229
B1	0	-5	Full rotation CCW	231
C	270	5	Partial: from 30 to 170 degrees	78
